# Epidemiology of Patients With Chronic Liver Disease Presenting to Emergency Departments in Australia

**DOI:** 10.1111/1742-6723.70310

**Published:** 2026-07-16

**Authors:** Akmez Latona, Katherine Stuart, Patricia Valery, Alan Ho, Biswadev Mitra

**Affiliations:** ^1^ Department of Emergency Medicine, Ipswich Hospital Ipswich Queensland Australia; ^2^ School of Public Health and Preventive Medicine Monash University Melbourne Victoria Australia; ^3^ Faculty of Medicine University of Queensland Brisbane Queensland Australia; ^4^ Department of Gastroenterology and Hepatology, Princess Alexandra Hospital Brisbane Queensland Australia; ^5^ QIMR Berghofer Herston Queensland Australia; ^6^ Queensland Cyber Infrastructure Foundation Brisbane Queensland Australia; ^7^ Emergency & Trauma Centre, Alfred Hospital Melbourne Victoria Australia

**Keywords:** chronic liver disease, decompensation, healthcare utilisation, liver cirrhosis, mortality

## Abstract

**Objective:**

To describe the epidemiology, healthcare utilisation and outcomes of patients presenting to emergency departments (EDs) with chronic liver disease (CLD) in Queensland, Australia.

**Methods:**

This statewide data linkage study included adult patients with CLD‐related diagnoses across 104 Queensland Health EDs between 1 January 2016 and 31 August 2023. Emergency, inpatient and mortality data were linked. Patients were stratified by cirrhosis status and decompensation. Outcome was 30‐day mortality. Poisson regression assessed trends, and Cox regression evaluated mortality.

**Results:**

Amongst 15,999,186 ED presentations, 23,578 (0.15%) were related to CLD, involving 11,961 patients. Presentations increased by 2% annually (IRR 1.02, 95% CI 1.02–1.03). Cirrhosis accounted for 18,735 presentations (79.5%). Overall, 20,312 presentations (86.1%) resulted in hospital admission, 918 (4.5%) were admitted to intensive care units (ICU), and 963 (4.1%) resulted in in‐hospital death. Amongst patients with cirrhosis, 16,968 (90.6%) resulted in admission, 867 (5.1%) were admitted to ICU and 899 (4.8%) died in hospital. Predictors of 30‐day mortality included cirrhosis (adjusted hazard ratio (aHR) 6.92, 95% CI 5.36–8.94), malignancy (aHR 3.21, 95% CI 2.90–3.55), hepatorenal syndrome (aHR 3.15, 95% CI 2.72–3.66), encephalopathy (aHR 2.03, 95% CI 1.78–2.32) and spontaneous bacterial peritonitis (aHR 1.47, 95% CI 1.20–1.80). Presentation to tertiary hospitals was associated with lower mortality (aHR 0.75, 95% CI 0.68–0.82).

**Conclusions:**

CLD‐related ED presentations are increasing and place substantial demand on hospital services in Queensland. Decompensation events strongly predict mortality and healthcare utilisation. ED‐initiated risk stratification and coordinated care models to improve outcomes for patients with cirrhosis require development and evaluation.

## 1 Introduction

Chronic Liver Disease (CLD) is a major global public health burden [1]. In Australia, more than six million people are affected, with healthcare costs exceeding $5.4 billion annually [2]. Alcohol remains the leading cause, although metabolic‐associated fatty liver disease (MAFLD), driven by obesity and Type 2 diabetes, is increasingly an important contributor [3, 4]. Hepatitis B virus (HBV) and hepatitis C virus (HCV) infections remain important global causes of CLD, although in some high‐income countries their overall incidence and prevalence have declined [5, 6]. Liver cirrhosis represents the advanced stage of CLD and carries high morbidity and mortality. Hospital admissions associated with cirrhosis have risen substantially in the last decade, particularly in Queensland, Australia [7]. The healthcare costs for cirrhosis exceed those of heart failure (HF) and chronic obstructive pulmonary disease (COPD), potentially driven by frequent emergency department (ED) presentations, prolonged hospital stays and increased resource use, with markedly higher costs during decompensation [8, 9, 10].

Patients often remain asymptomatic during the compensated phase; however, progression to decompensated cirrhosis is marked by complications including ascites, hepatic encephalopathy and gastrointestinal haemorrhage from portal hypertension. Acute decompensation may be further complicated by acute‐on‐chronic liver failure (ACLF), a syndrome associated with extrahepatic organ failure and high short term mortality [11]. These complications frequently require emergency care and contribute substantially to hospitalisation and healthcare utilisation [12].

Patients with cirrhosis presenting to the ED often have features of decompensation. Factors such as high model for end‐stage liver disease‐sodium (MELD‐Na) score and prior ED encounters are associated with higher ED utilisation [13]. Common ED presentations include abdominal pain, sepsis and gastrointestinal haemorrhage. Mortality following an ED visit is higher in the setting of cirrhosis, with a 90‐day mortality rate more than double that of HF and COPD [14]. However, current trends in CLD‐related ED presentations and ED utilisation patterns in Australia remain underexplored.

The aims of this study were to describe the demographics, clinical characteristics and outcomes of patients presenting to Queensland Health EDs with a CLD‐related diagnosis. The secondary objective was to identify subgroups with greater healthcare utilisation and mortality. We hypothesised that patients with diagnosed cirrhosis and decompensation would exhibit worse outcomes.

## 2 Methodology

### 2.1 Setting

This was a retrospective cohort study using linked emergency, inpatient and mortality data across all 104 Queensland Health EDs between 1 January 2016 and 31 August 2023.

### 2.2 Databases and Data Linkage

We utilised data from the Emergency Data Collection (EDC), Queensland Health Admitted Patient Data Collection (QHADPC), and Death Registry to track patient journeys from emergency presentation to hospital discharge or death, using established data linkage methods that have been previously described [15].

### 2.3 Cohort

Adult patients who presented to any Queensland Health EDs with a principal or additional diagnosis related to CLD were included. The study cohort was identified from the EDC. All cohort definitions and classifications were based on International Classification of Diseases, 10th Revision, Australian Modification (ICD‐10‐AM) codes (Supporting Information) [16, 17, 18, 19].

Patients were categorised by the presence or absence of cirrhosis. Presentations with cirrhosis were further stratified according to the presence of decompensation and those with further decompensation. Decompensation was defined as variceal haemorrhage, ascites or encephalopathy [11]. Further decompensation was defined as the presence of hepatorenal syndrome (HRS), spontaneous bacterial peritonitis (SBP) or jaundice in patients with decompensation [20].

### 2.4 Aetiology and Procedures

Aetiologies and cofactors were categorised into alcohol, viral, MAFLD (including non‐alcoholic steatohepatitis, non‐alcoholic fatty liver disease), metabolic (haemochromatosis, Wilson disease and alpha‐1 antitrypsin deficiency), autoimmune (primary biliary cholangitis, primary sclerosing cholangitis and autoimmune hepatitis), inflammatory and hepato‐occlusive disorders. Procedures associated with cirrhosis included banding, ligation or coiling of oesophageal or gastric varices, paracentesis, trans‐jugular intrahepatic porto‐systemic shunt placement (TIPS).

### 2.5 Socioeconomic Factors

These were based on the patient's place of residence EDs and were classified according to the Accessibility/Remoteness Index of Australia (ARIA+) as Major Cities (highly accessible), Regional (inner and outer regional areas) or Remote/Very Remote. Using the Socio‐Economic Indexes for Areas (SEIFA), place of residence was classified into five quintiles, Q1 to Q5, with Q1 representing the most affluent and Q5 the most disadvantaged areas.

### 2.6 Hospital Factors

Hospitals were classified as tertiary versus non‐tertiary, based on the presence of on‐site hepatology care. The Australasian Triage Scale defined urgency of ED presentation, from Category 1 (immediate), Category 2 (within 10 min), Category 3 (30 min), Category 4 (60 min) and Category 5 (120 min) [21]. In‐hours presentations were classified as 08:00–18:00 h, and after‐hours as 18:00–08:00 h.

### 2.7 Outcomes

We examined 30‐day mortality and healthcare utilisation using hospital length of stay (LOS) for the overall CLD cohort and subgroups based on presence/absence of cirrhosis, decompensation and further decompensation.

### 2.8 Data Analysis

Categorical variables were summarised as frequencies and percentages; continuous variables as means with standard deviations (SD) or medians with interquartile range (IQR). Annual trends in CLD‐related ED presentations were described using age‐standardised rates per 100,000 adult Queensland population, directly standardised to the Australian standard population, with 95% confidence intervals calculated using the Dobson method [22]. Poisson regression was used to assess trends using the adult Queensland population and total adult ED presentations as offsets, representing community burden and ED workload, respectively. Results were reported as incidence rate ratios (IRR) with 95% confidence intervals. Associations between categorical variables were assessed using chi‐squared or Fisher's exact tests.

For ED presentation‐based analysis, predictors of LOS were assessed using linear mixed‐effects regression to account for repeated ED presentations per patient. Multivariate models used backward elimination for selection of variables for inclusion in the model.

For patient‐based analysis, 30‐day survival was evaluated from each patient's final ED presentation within the study period, using Kaplan–Meier analysis and Cox proportional hazards regression, with proportional hazards tested using Schoenfeld residuals. Hazard ratios (HR) with 95% confidence intervals were reported. Variables for the multivariable analysis were selected using backward stepwise elimination, and results were reported as adjusted hazard ratios (aHR).

All analyses were performed using R software 4.4.1, with statistical significance set at 0.05. The study was approved by Metro South Human Research Ethics Committee (HREC/102281), with the requirement to seek informed consent from patients waived.

## 3 Results

### 3.1 Overall CLD Cohort

**FIGURE 1:**
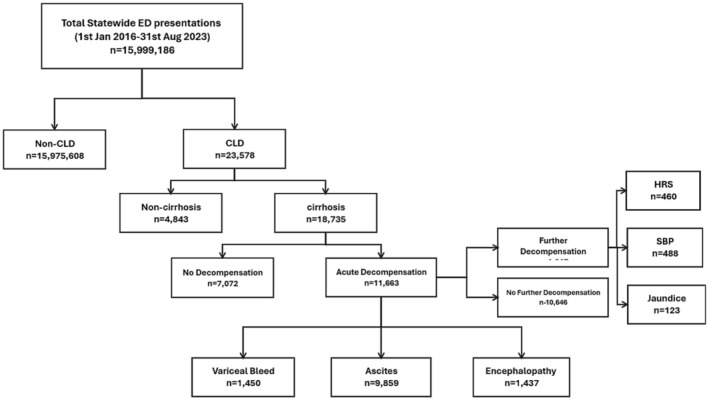
Flow diagram of study population and sub‐categories. CLD, chronic liver disease; ED, emergency department; HRS, hepatorenal syndrome; SBP, spontaneous bacterial peritonitis.

Figure 1 illustrates the study flow diagram, outlining cohort selection and subgroup categorisation. Over the study period, there were 15,999,186 all‐cause adult ED presentations to QH facilities, of which 36.2% resulted in hospitalisation, 0.23% required ICU admission with an all‐cause in‐hospital mortality rate of 0.24%. CLD‐related ED presentations accounted for 23,578 cases (0.15%) involving 11,961 individual patients, with 8156 patients presenting once, 1765 presenting twice and 2040 presenting three or more times to ED.

**FIGURE 2:**
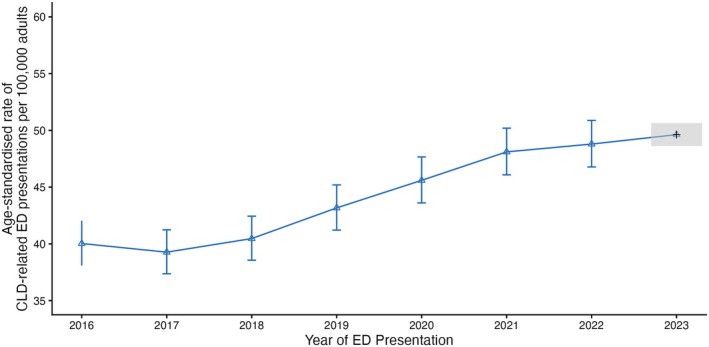
Age‐standardised rates of CLD‐related ED presentations per 100,000 adult Queensland population. The 2023 rate was estimated using a generalised additive model based on data available to August 2023.

**FIGURE 3:**
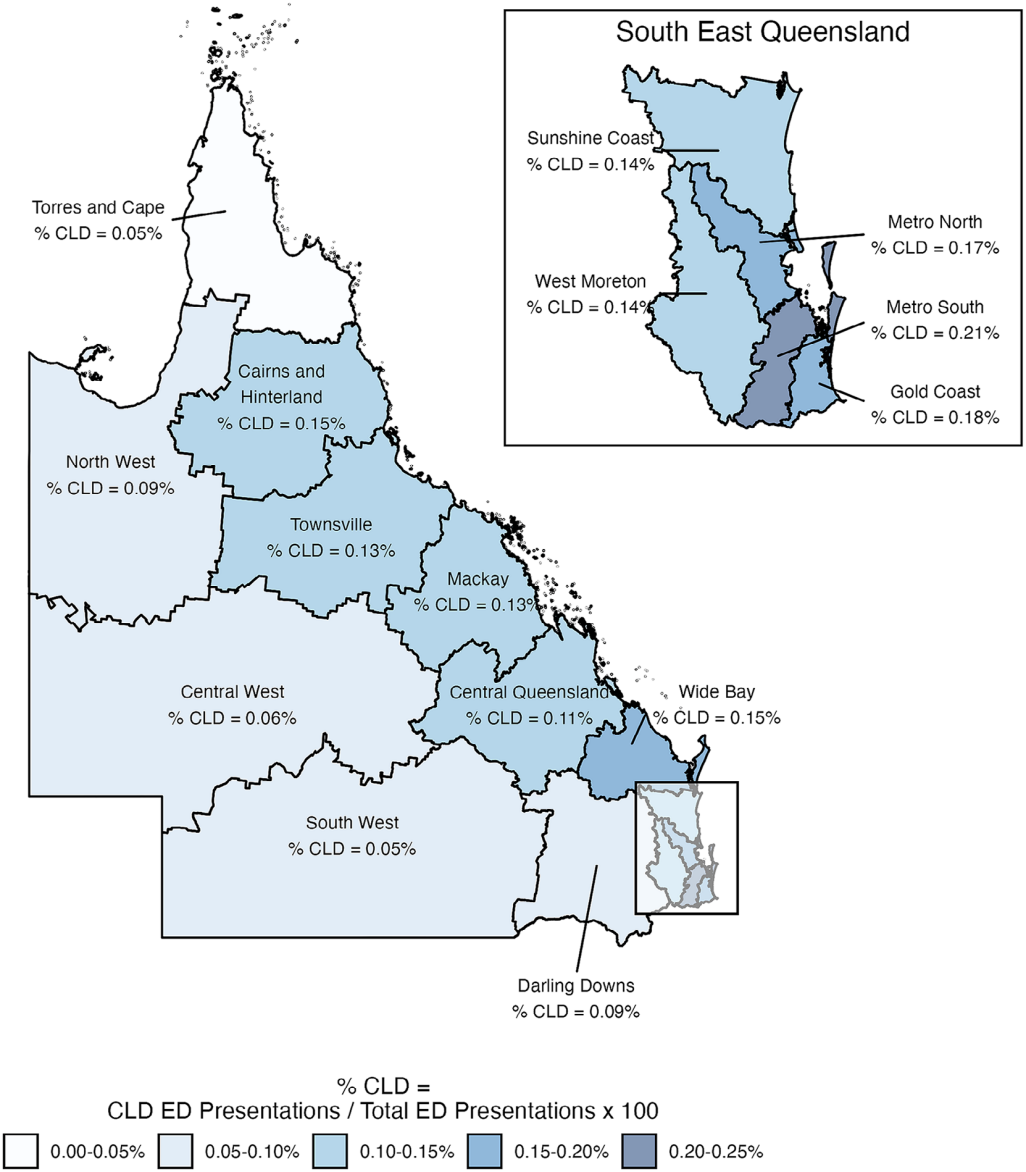
Heatmap showing geographic distribution of CLD‐related ED presentations according to Queensland Hospital Health Service.

Figure 2 shows age‐standardised rates of CLD‐related ED presentations per 100,000 adult Queensland population. Rates increased from 40.0 (95% CI 38.1–42.0) in 2016 to 48.8 (95% CI 46.8–50.9) in 2022. When adjusted for total adult ED presentations, CLD‐related ED presentations increased on average 2% per year from 2016 to 2023 (IRR = 1.02, 95% CI: 1.02–1.03, *p* < 0.001) for both patients with cirrhosis (IRR 1.02, 95% CI 1.01–1.02, *p* = 0.001) and more sharply for patients without cirrhosis (IRR 1.07, 95% CI: 1.06–1.08, *p* < 0.001) (Figure S1). Of the whole state, Metro South and Metro North Hospital and Health Services, which cover the areas south and north of the Brisbane River respectively, recorded the highest proportions of CLD‐related ED presentations (20.7% and 19.1%, respectively). In contrast, Central West and Torres Cape HHS had the lowest proportions (0.2% and 0.4%, respectively) (Figure 3).

Presentations were concentrated in patients who resided in major cities (53.4%), followed by regional areas (39.4%) and rural/remote areas (3.2%) based on ARIA scores. SEIFA scores showed that 44.6% of presentations were from patients residing in disadvantaged areas, 26.9% from average areas, 8.7% from advantaged areas and 19.8% from unspecified locations.

Non‐tertiary EDs received 53.4% of CLD‐related presentations, and 33.0% presented after hours. The majority of patients (54.4%) arrived via ambulance, 44.4% were ambulatory and 1.2% by aerial retrieval (133 by helicopter and 146 by fixed‐wing aircraft). Of the CLD‐related presentations, 64% were triaged as ATS Category 3, 17% as Category 2 and 1.8% as Category 1 (Table 1).

**TABLE 1:** Demographics and clinical characteristics of CLD‐related ED presentations according to cirrhosis status.

Characteristics (*N*, %)	Cirrhosis	Non‐Cirrhosis	Total	*p* ^a^
Sample size (*N*)	18,735	4843	23,578	
Patient factors				
Age distribution (years)				< 0.001
< 40	1537 (8.2%)	1312 (27%)	2849 (12%)	
40–59	9281 (50%)	2106 (43%)	11,387 (48%)	
60–79	7062 (38%)	1200 (25%)	8262 (35%)	
≥ 80	855 (4.6%)	225 (4.6%)	1080 (4.6%)	
Biological sex, male	12,251 (65%)	2706 (56%)	14,957 (63%)	< 0.001
First Nations peoples	1831 (9.8%)	288 (5.9%)	2119 (9.0%)	< 0.001
Remoteness of residence (ARIA)				< 0.001
Major City	9996 (53.4%)	2586 (53.4%)	12,582 (53.4%)	
Regional	7442 (39.7%)	1853 (38.3%)	9295 (39.4%)	
Remote/very remote	584 (3.1%)	182 (3.8%)	766 (3.2%)	
Missing	713 (3.8%)	222 (4.6%)	935 (4.0%)	
Socioeconomic status (SEIFA)				< 0.001
Q1	1609 (8.6%)	443 (9.1%)	2052 (8.7%)	
Q2	2149 (11.5%)	606 (12.5%)	2755 (11.7%)	
Q3	2805 (15.0%)	887 (18.3%)	3692 (15.7%)	
Q4	3359 (17.9%)	1206 (24.9%)	4565 (19.4%)	
Q5	4792 (25.6%)	1164 (24.0%)	5956 (25.3%)	
Missing	3778 (20.2%)	890 (18.4%)	4668 (19.8%)	
Presumed aetiology/cofactor^b^				
Alcohol	11,411 (61%)	1298 (27%)	12,709 (54%)	< 0.001
Viral	3109 (17%)	848 (18%)	3957 (17%)	0.13
Metabolic	1765 (9.4%)	88 (1.8%)	1853 (7.9%)	< 0.001
MAFLD	1123 (6.0%)	500 (10%)	1623 (6.9%)	< 0.001
Autoimmune	345 (1.8%)	176 (3.6%)	521 (2.2%)	< 0.001
Inflammatory	99 (0.5%)	949 (20%)	1048 (4.4%)	< 0.001
Budd Chiari	43 (0.2%)	1 (< 0.1%)	44 (0.2%)	0.003
CCI category				< 0.001
0	82 (0.4%)	486 (10%)		
1	1123 (6.0%)	1805 (37%)		
2	1279 (6.8%)	952 (20%)		
≥ 3	16,251 (87%)	1600 (33%)		
Comorbidities				
T2DM	4668 (25%)	382 (7.9%)	5050 (21%)	< 0.001
Obesity	2103 (11%)	302 (6.2%)	2405 (10%)	< 0.001
Malignancy	2369 (13%)	211 (4.4%)	2580 (11%)	< 0.001
CKD	1139 (6.1%)	80 (1.7%)	1219 (5.2%)	< 0.001
HF	919 (4.9%)	84 (1.7%)	1003 (4.3%)	< 0.001
COPD	438 (2.3%)	66 (1.4%)	504 (2.1%)	< 0.001
PUD	253 (1.4%)	8 (0.2%)	261 (1.1%)	< 0.001
CVD	117 (0.6%)	16 (0.3%)	133 (0.6%)	0.015
MI	81 (0.4%)	25 (0.5%)	106 (0.4%)	0.4
PVD	81 (0.4%)	12 (0.2%)	93 (0.4%)	0.068
Hospital factors				
Tertiary hospitals	8923 (48%)	2060 (43%)	10,983 (47%)	< 0.001
In‐hours presentation	12,621 (67%)	3180 (66%)	15,801 (67%)	0.025
ICU admission	867 (4.6%)	51 (1.1%)	918 (3.9%)	< 0.001
ATS category				< 0.001
1	372 (2.0%)	43 (0.9%)	415 (1.8%)	
2	3343 (18%)	732 (15%)	4075 (17%)	
3	12,180 (65%)	2921 (60%)	15,101 (64%)	
4	2424 (13%)	984 (20%)	3408 (14%)	
5	416 (2.2%)	163 (3.4%)	579 (2.5%)	
Liver factors				< 0.001
Decompensation	11,663 (62%)	0 (0%)	11,663 (49%)	
Ascites	9859 (53%)	0 (0%)	9859 (42%)	
Variceal haemorrhage	1450 (7.7%)	0 (0%)	1450 (6.1%)	
Hepatic encephalopathy	1437 (7.7%)	0 (0%)	1437 (6.1%)	
Jaundice	283 (1.5%)	0 (0%)	283 (1.2%)	
SBP	571 (3.0%)	0 (0%)	571 (2.4%)	
HRS	636 (3.4%)	0 (0%)	636 (2.7%)	

Abbreviations: ARIA: Accessibility/Remoteness Index of Australia, ATS: Australasian Triage Scale, CCF: Congestive Heart Failure, CCI: Charlson Comorbidity Index, CKD: Chronic Kidney Disease, CLD: Chronic Liver Disease, COPD: Chronic Obstructive Pulmonary Disease, CVD: Cerebrovascular Disease, ED: Emergency Department, HRS: Hepatorenal Syndrome, MI: Myocardial Infarct, PUD: Peptic Ulcer Disease, PVD: Peripheral Vascular Disease, SBP: Spontaneous Bacterial Peritonitis, SEIFA: Socio‐Economic Indexes for Areas, T2DM: Type 2 diabetes mellitus.

^a^ Pearson's chi‐squared test; Fisher's exact test.

^b^ Patients may have more than one presumed aetiology/cofactor.

Amongst all 23,578 CLD‐related ED presentations, 20,312 (86.1%) resulted in hospital admission, 2312 (11.4%) were discharged, 818 (4.0%) were transferred to another hospital and 60 (0.3%) died in the ED.

Of those admitted, mean hospital LOS was 5.6 days (SD = 7.2). There were 918 (4.5%) presentations admitted to ICU; the mean ICU LOS was 4.5 days (SD = 6.1) and mean duration of mechanical ventilation was 5.6 days (SD = 6.7). In‐hospital mortality was 4.1% (963 deaths).

### 3.2 Presentations Stratified by Cirrhosis Status

Amongst CLD‐related ED presentations, 18,735 (79.5%) had a diagnosis of cirrhosis. Compared to patients without cirrhosis (4843), cirrhosis‐related presentations were more often male (65% vs. 56%, *p* < 0.001), aged > 40 years (92.6% vs. 72.6%, *p* < 0.001) and identified as First Nations people (9.8% vs. 5.9%, *p* < 0.001). Alcohol‐related liver disease was predominant in the cirrhosis cohort (61% vs. 27%, *p* < 0.001), whilst MAFLD (6.0% vs. 10%, *p* < 0.001) and autoimmune diseases (1.8% vs. 3.6%, *p* < 0.001) were more common in non‐cirrhosis. Cirrhosis related ED presentations had a heavier comorbidity burden, reflected by a greater proportion having a Charlson Comorbidity Index (CCI) score ≥ 3 (87% vs. 33%, *p* < 0.001). Cirrhosis related ED presentations were more frequent in tertiary hospitals (48% vs. 43%, *p* < 0.001) and a greater proportion were ATS Category 1 compared to presentations without cirrhosis (2.0% vs. 0.9%, *p* < 0.001). Non‐variceal gastrointestinal bleeding was more frequent in cirrhosis than in presentations without cirrhosis (5.6% vs. 1.8%, *p* < 0.001).

**FIGURE 4:**
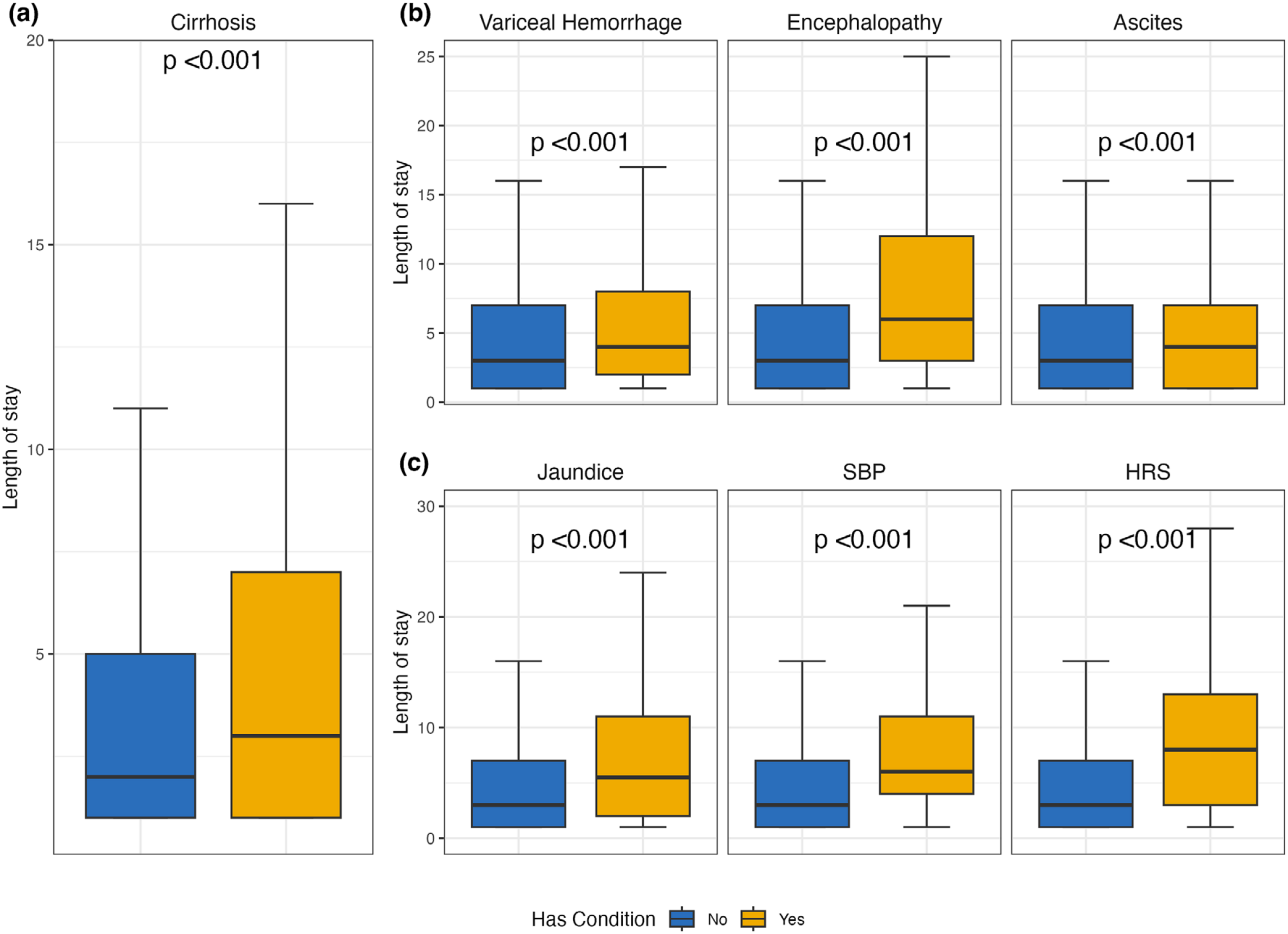
Length of hospital stay (days) according to (a) cirrhosis status, (b) acute decompensation event and (c) further decompensation event.

Of the 18,735 cirrhosis‐related ED presentations, 16,968 (90.6%) resulted in hospital admission, 948 (5.1%) were discharged, 712 (3.8%) were transferred to another hospital and 56 (0.3%) died in ED. Of those admitted, the mean hospital LOS was 4.1 days (SE = 0.14) and 867 (5.1%) were admitted to ICUs, with a mean ICU LOS of 3.7 days (SE = 0.90) and mean duration of mechanical ventilation of 5.16 days (SE = 1.51). In‐hospital mortality of ED presentations with cirrhosis was 4.8% (899 deaths). Cirrhosis was associated with prolonged hospitalisation, with longer mean LOS by 2.1 days (95% CI: 1.8–2.4, *p* < 0.001) compared to non‐cirrhosis (Table 2, Figure 4).

**TABLE 2:** Impact of liver factors on hospital length of stay.

Characteristic	Unadjusted			Adjusted^a^		
	Change in LOS	95% CI	*p*	Change in LOS	95% CI	*p*
Presentations with CLD (*n* = 20,312)						
Cirrhosis	2.1	1.8–2.4	< 0.001			
Presentations with Cirrhosis (*n* = 17,450)						
Acute decompensation						
Variceal haemorrhage	0.92	0.49–1.4	< 0.001	1.3	0.90–1.7	< 0.001
Encephalopathy	3.6	3.2–4.0	< 0.001	3.3	2.9–3.7	< 0.001
Ascites	1.1	0.87–1.3	< 0.001	1.1	0.84–1.3	< 0.001
Further decompensation						
Jaundice	3.3	2.4–4.1	< 0.001	3.5	2.6–4.3	< 0.001
SBP	4.9	4.3–5.5	< 0.001	4.1	3.6–4.7	< 0.001
HRS	6.5	5.9–7.1	< 0.001	5.9	5.3–6.4	< 0.001

Abbreviations: CI: confidence interval; CLD: chronic liver disease; HRS: hepatorenal syndrome; LOS: length of stay.

^a^ Multivariable model included variceal haemorrhage, encephalopathy, ascites, jaundice, peritonitis and HRS using backwards elimination. Cirrhosis was excluded as the adjusted model was limited to patients with cirrhosis, and its inclusion alongside decompensation features would introduce multicollinearity.

### 3.3 Presentations With Decompensation

Amongst the cirrhosis cohort, 11,663 (62.3%) presented with decompensation, which was associated with a higher proportion of ATS Category 1 (*p* < 0.001). Ascites was the most frequent decompensation event (9859, 84.5%). Further decompensation developed during admission in 1017 presentations (8.7%), with SBP reported in 488 (48%) and HRS in 460 (45%). Procedures in the cirrhosis cohort included paracentesis (6972, 37.2%), ligation of varices (464, 2.5%) and TIPS (8, 0.1%).

The majority of ED presentations with decompensation (11,663) resulted in hospital admission (10,827, 92.8%), whilst only 369 (3.2%) were discharged, 430 (3.7%) were transferred to another hospital and 16 (0.1%) died in ED. Of those admitted, the median hospital LOS was 4.0 days (IQR 2.0–8.0) and 492 (4.2%) required ICU care, with a median ICU LOS of 2.7 days (IQR 1.1–7.4) and median duration of mechanical ventilation of 4.3 days (IQR 1.9–8.0). In‐hospital mortality amongst ED presentations with decompensation was 514 (4.4%).

Decompensation was associated with significantly longer hospital LOS and lower 30‐day survival compared to cirrhosis ED presentations without decompensation (Table 2, Figure 4). Encephalopathy had the greatest impact on hospital LOS, prolonging admission by a mean of 3.3 days (95% CI: 2.9–3.8), followed by variceal haemorrhage (+1.3 days, 95% CI: 0.9–1.7, *p* < 0.001). Further decompensation during admission resulted in higher hospital LOS, with an additional 5.9 days for HRS (95% CI: 5.3–6.4, *p* < 0.001) and 4.1 days for SBP (95% CI: 3.6–4.7, *p* < 0.001).

### 3.4 30‐Day Survival From Last ED Presentation

**FIGURE 5:**
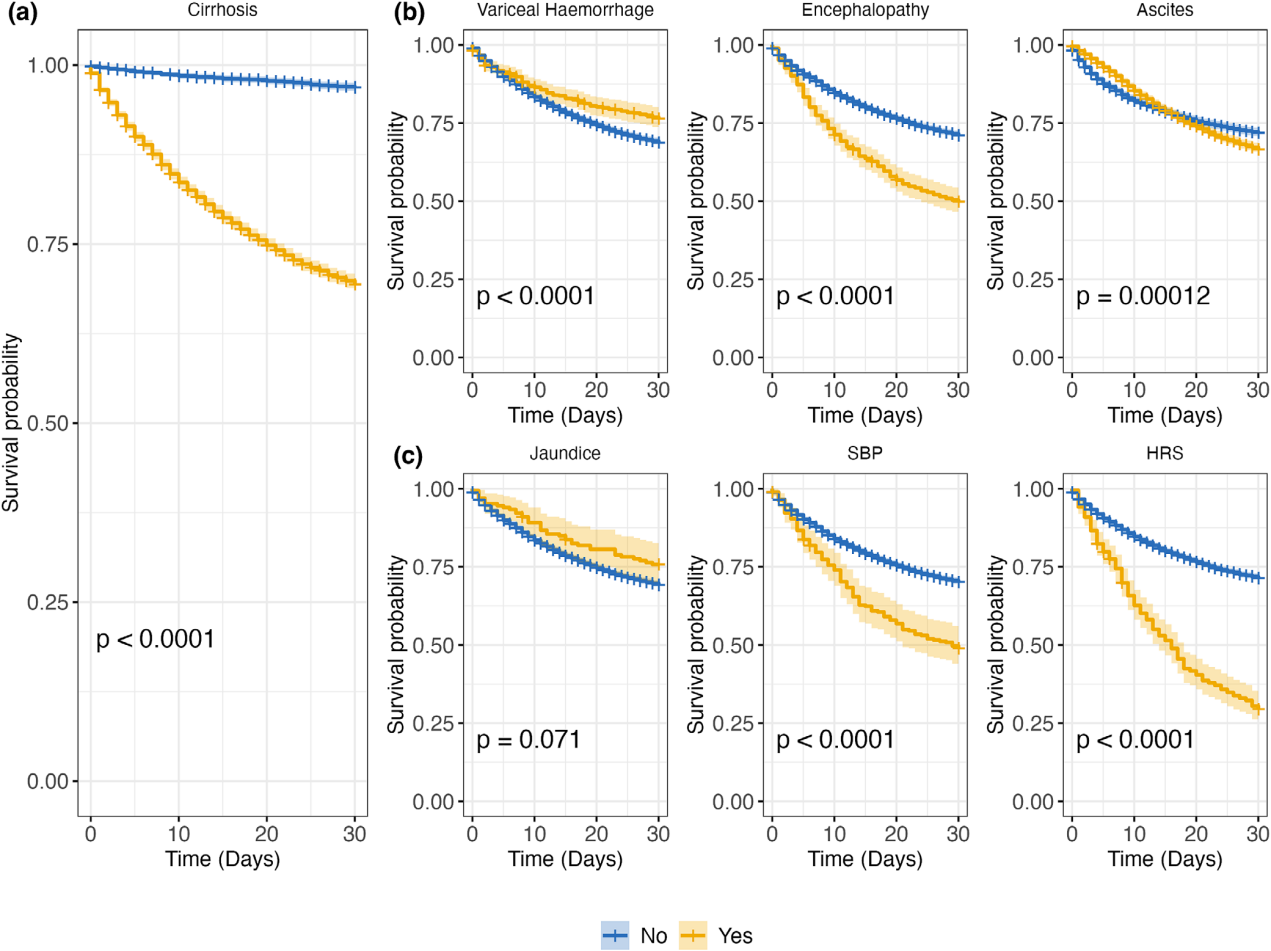
Probability of 30‐day survival by (a) cirrhosis status, (b) variceal haemorrhage, encephalopathy, and ascites, and (c) jaundice, spontaneous bacterial peritonitis (SBP), and hepatorenal syndrome (HRS).

The overall 30‐day survival probability was 78.0% (95% CI: 77.3–78.8) (Table 3), with a 30‐day mortality of 22.0%. Amongst those with cirrhosis, the 30‐day survival probability was 69.4% (95% CI: 68.4–70.4), with a 30‐day mortality of 30.6%. Kaplan–Meier curves for cirrhosis and decompensation events are shown in Figure 5. Univariable analyses of variables associated with 30‐day mortality are listed in Table 4. In multivariable model, older age (80+ years vs. < 40 years aHR = 2.31, 95% CI: 1.76–3.03, *p* < 0.001) and male sex (aHR 1.15, 95% CI: 1.05–1.27, *p* = 0.004) were independently associated with 30‐day mortality. Cirrhosis was also associated with 30‐day mortality (aHR 6.92, 95% CI: 5.36–8.94, *p* < 0.001), along with malignancy (aHR 3.21, 95% CI: 2.90–3.55, *p* < 0.001). Amongst decompensation events, HRS (aHR 3.15, 95% CI: 2.72–3.66, *p* < 0.001), encephalopathy (aHR 2.03, 95% CI: 1.78–2.32, *p* < 0.001) and SBP (aHR 1.47, 95% CI: 1.20–1.80, *p* < 0.001) were associated with 30‐day mortality. Presentation to a tertiary hospital was associated with lower 30‐day mortality (aHR 0.75, 95% CI: 0.68–0.82, *p* < 0.001).

**TABLE 3:** Probability of patient survival at 0, 15 and 30 days since last ED presentation.

Days since last ED presentation	Number at risk	Survival probability (%)	95% CI
0	11,961	99.2	99.0–99.3
15	10,067	84.8	84.1–85.4
30	9112	78.0	77.3–78.8

**TABLE 4:** Impact of patient and hospital factors on 30‐day mortality in patients with a diagnosis of CLD recorded at their latest ED presentation.

Characteristic	Unadjusted		Adjusted	
	HR (95% CI)	*p*	aHR (95% CI)	*p*
Sociodemographic factors				
Age category (years)				
< 40	Reference		Reference	
40–59	2.97 (2.35, 3.76)	< 0.001	1.53 (1.21, 1.94)	< 0.001
60–79	4.63 (3.67, 5.83)	< 0.001	1.89 (1.49, 2.39)	< 0.001
80+	5.57 (4.28, 7.25)	< 0.001	2.31 (1.76, 3.03)	< 0.001
Gender				
Female	Reference		Reference	
Male	1.37 (1.25, 1.51)	< 0.001	1.15 (1.05, 1.27)	0.004
First nations				
No	Reference		N/S	
Yes	0.80 (0.67, 0.95)	0.012		
ARIA				
Major city	Reference		N/S	
Regional/Remote/Very remote	1.23 (1.12, 1.34)	< 0.001		
SEIFA				
Most affluent (Q1/Q2/Q3)	Reference		Reference	
Most disadvantaged (Q4/Q5)	1.29 (1.18, 1.41)	< 0.001	1.09 (0.99, 1.20)	0.073
Clinical factors				
Alcohol	1.18 (1.08, 1.29)	< 0.001	1.18 (1.07, 1.31)	0.001
Type II Diabetes	1.24 (1.12, 1.37)	< 0.001	0.83 (0.75, 0.93)	0.001
Obesity	0.95 (0.83, 1.10)	0.5	0.88 (0.76, 1.02)	0.082
Malignancy	3.58 (3.27, 3.93)	< 0.001	3.21 (2.90, 3.55)	< 0.001
CKD	2.19 (1.91, 2.50)	< 0.001	1.55 (1.34, 1.79)	< 0.001
CCF	1.70 (1.47, 1.98)	< 0.001	1.27 (1.08, 1.48)	0.003
COPD	1.69 (1.36, 2.09)	< 0.001	1.28 (1.03, 1.59)	0.027
Cirrhosis	11.4 (8.89, 14.5)	< 0.001	6.92 (5.36, 8.94)	< 0.001
Decompensation events				
Ascites	1.90 (1.73, 2.07)	< 0.001	0.93 (0.85, 1.02)	0.12
Variceal Haemorrhage	1.05 (0.86, 1.28)	0.6	N/S	
Encephalopathy	2.86 (2.52, 3.25)	< 0.001	2.03 (1.78, 2.32)	< 0.001
Jaundice	0.97 (0.69, 1.37)	0.9	N/S	
SBP	2.70 (2.22, 3.29)	< 0.001	1.47 (1.20, 1.80)	< 0.001
HRS	4.57 (3.97, 5.26)	< 0.001	3.15 (2.72, 3.66)	< 0.001
Hospitals factors				
Non‐tertiary	Reference		Reference	
Tertiary	0.78 (0.72, 0.86)	< 0.001	0.75 (0.68, 0.82)	< 0.001
In‐hours presentation				
In	Reference		N/S	
After	1.00 (0.91, 1.10)	> 0.9		
Transfer to another hospital	1.39 (1.10, 1.75)	0.006	1.25 (0.98, 1.58)	0.067

*Note:* Eleven thousand nine hundred sixty‐one patients with 23,578 presentations.

Abbreviations: ARIA: Accessibility/Remoteness Index of Australia, CCF: Congestive cardiac failure, CI: Confidence interval, CKD: Chronic kidney disease, COPD: Chronic obstructive pulmonary disease, HR: Hazard ratio, HRS: Hepatorenal syndrome, N/S: Not selected (during backwards elimination), SBP: Spontaneous bacterial peritonitis, SEIFA: Socio‐Economic Indexes for Areas.

## 4 Discussion

This study provides the first characterisation of CLD‐related ED presentations across a statewide population of Australasia, offering an ED perspective on outcomes and healthcare utilisation. CLD‐related presentations increased over the study period and placed a disproportionate burden on emergency and inpatient services compared to the general ED population, with higher rates of hospitalisation, ICU admission and 30‐day deaths. Age‐standardised rates demonstrated an increasing population burden of CLD‐related ED presentations over time, whilst analyses adjusted for total adult ED presentations showed that CLD accounted for an increasing proportion of overall ED workload. These findings suggest that the observed increase extends beyond population growth and demographic change, reflecting a growing demand on emergency care services. One‐fifth of CLD‐related ED presentations were classified as ATS Category 1 or 2, reflecting high clinical acuity. Cirrhosis was associated with the majority of CLD‐related ED presentations and in turn was strongly predictive of worse outcomes including higher ICU admissions, longer hospitalisations and significantly reduced 30‐day survival compared to presentations without cirrhosis. The inclusion of the non‐cirrhosis cohort provided an epidemiological comparator demonstrating the substantially greater healthcare utilisation and mortality associated with cirrhosis and decompensation. Amongst patients presenting with acute decompensation, hepatic encephalopathy was the strongest predictor of death, whilst further decompensation events—HRS and SBP—were associated with poor outcomes. These findings underscore the substantial burden of cirrhosis in the ED setting and identify high‐risk subgroups who may benefit from early risk stratification and targeted care pathways.

The majority of CLD‐related ED presentations occurred in Metro North, Metro South and Gold Coast HHS—Queensland's most densely populated regions and locations of established tertiary hepatology services. Presentation to a tertiary hospital was associated with lower 30‐day mortality, potentially reflecting ready access to specialist hepatology, haematology services and accompanying surgical, ICU and interventional radiology resources. The finding that after‐hours ED presentation or interhospital transfer were not significantly associated with 30‐day mortality may reflect the Queensland Health ED care network, specifically its effective local capabilities, coordinated referral and retrieval systems that support equitable patient outcomes across a broad range of geographic settings [23, 24, 25]. Ongoing attention to access and timeliness in remote/regional areas remains important. In a recent study, patients with decompensated cirrhosis managed in a hepatologist‐led coordinated care model at two Australian tertiary hospitals had fewer liver‐related emergency re‐admissions and improved survival compared to standard care [26]. Future research should prospectively evaluate ED‐initiated hepatology referral models and coordinated care pathways across metropolitan and regional settings in Queensland.

Variceal haemorrhage was not independently associated with increased 30‐day mortality. Over one‐third of patients with variceal bleeding were transferred to another hospital. The absence of a mortality signal reflects early recognition, appropriate resuscitation and retrieval services carrying blood products, enabling timely access to definitive care—including medical treatments and therapeutic endoscopy—facilitated by Queensland's coordinated retrieval and inter‐hospital transfer network. This finding aligns with a study by Powell et al., which reported reduced odds of in‐hospital mortality (OR = 0.68) for variceal bleeding in patients with cirrhosis hospitalised in Queensland, although the focus of this study was on admitted inpatients rather than ED presentations [27]. In contrast, other studies have reported higher mortality associated with variceal bleeding (OR = 1.54) [28]. Differences in system‐level factors—particularly in access to acute interventions, retrieval services and endoscopic care—explain variation in outcomes across jurisdictions [28].

In our study, in‐hospital mortality for cirrhosis‐related ED presentations was 4.8%. These figures vary from prior ED‐based cohorts. A multi‐centre study by Javaud et al. across three EDs in France (609 visits, 224 patients) reported a 30‐day mortality of approximately 25%, whilst Ximenes et al. reported an in‐hospital mortality of 25% in a single ED cohort in Brazil (277 admissions, 149 patients) [29, 30]. In contrast, Parvataneni et al. reported a 9% in‐hospital mortality amongst 2213 ED presentations across 16 hospitals in Indiana, United States [13]. Several factors may explain this variation. Our study spans 7.5 years and includes more than 100 EDs across metropolitan, regional, rural and remote Queensland—a geographically vast state covering over 1.8 million square kilometres with a population of approximately 5 million—capturing a heterogeneous population and broader case mix than single‐centre or predominantly metropolitan studies. This wider geographic and system‐level representation introduces diversity in demographics, disease severity, access to specialist services and models of care, all of which influence case mix and outcomes. In univariate analysis, regional and remote residence and socioeconomic disadvantage were associated with higher 30‐day mortality; however, these associations were not retained after adjustment for clinical and hospital‐level factors. This suggests that the observed disparities are more likely driven by differences in disease severity, comorbidity burden and access to tertiary‐level services rather than the patient's place of residence (remoteness and socioeconomic status) independently. Similarly, Powell et al., in a cohort of admitted inpatients in public hospitals in Queensland, reported a disproportionate burden of cirrhosis amongst individuals residing in the most socioeconomically disadvantaged areas, with socioeconomic disadvantage associated with in‐hospital mortality in univariate but not multivariable analysis, consistent with the present findings [27]. An important clinical distinction not captured in this study was acute‐on‐chronic liver failure (ACLF), a syndrome associated with organ failure and high short‐term mortality [11]. As this was a statewide ICD‐10‐AM coding‐based linkage study, ACLF could not be reliably identified because established diagnostic criteria require detailed clinical and laboratory parameters not captured within administrative datasets. Future studies using granular clinical data are needed to better characterise ACLF presentations and outcomes in the ED setting.

In this study, the inclusion criteria for the CLD cohort were broadened to include ICD codes for cirrhosis as well as chronicity with the intent to capture both diagnosed and undiagnosed cases of cirrhosis, particularly in rural or remote areas of Queensland where there is limited access to hepatology care to make a formal diagnosis. Many patients with cirrhosis are diagnosed late, with up to 70% presenting with complications [31]. In the ED, the distinction between CLD and cirrhosis is less clinically relevant, as the focus is on managing complications and decompensation events. By interrogating the EDC, ED presentations and complications relevant to CLD alone are captured, rather than including everyone with a history of CLD who is admitted for non‐CLD‐related reasons and subsequently develops liver‐related complications. This approach aligns with the algorithm described by King et al., chosen to ensure consistency in future studies analysing coagulopathy and use of blood products in CLD‐related ED presentations [32]. In contrast, other studies, utilised more restrictive criteria notably only cirrhosis‐related codes or focused on hospital admissions rather than ED presentations [4, 13, 27].

Our study highlights the need for a risk‐stratification pathway initiated in the ED to identify patients with cirrhosis at increased risk of mortality. Gastroenterology Society of Australia has developed care bundles for the first 24 h of presentation. However, awareness and uptake within EDs remain limited [33]. These care bundles may be particularly useful in non‐tertiary centres without dedicated hepatology services. Huang et al. demonstrated that acute care bundles for decompensated cirrhosis were associated with improved 90‐day mortality irrespective of disease severity, although no significant differences were observed in readmission rates or long‐term survival. The study was conducted in a single‐centre metropolitan hospital in Brisbane, where access to multiple nearby tertiary hepatology referral centres may limit generalisability to broader healthcare networks and geographically dispersed populations [34]. Wigg et al. demonstrated in a multicentre randomised controlled trial that a chronic disease management model for decompensated cirrhosis reduced encephalopathy‐related emergency admissions despite no reduction in overall liver‐related emergency admissions. In our study, encephalopathy was associated with the greatest increase in LOS amongst decompensation, supporting it as a major contributor to healthcare utilisation and a potential target for intervention [35]. Prospective studies implementing care bundles are warranted to determine which components improve healthcare utilisation in Queensland's geographical context. Early ED implementation may support timely escalation and referral, particularly in regional and rural hospitals, enabling earlier engagement with tertiary hepatology services.

Data linkage used in our study has several limitations. The reliance on ICD codes for identifying clinical diagnosis may lack specificity and granularity, leading to potential misclassification or incomplete capture of disease characteristics. Variability in coding practises across institutions may introduce bias. As the dataset was provided in a de‐identified format through Data Linkage Queensland, independent verification of individual‐level linkage accuracy or participant selection by the research team was not possible. This is an inherent characteristic of population‐level data linkage studies. Some ICD codes do not account for chronicity. ICD‐10‐AM coding does not reliably distinguish refractory manifestations of decompensation, including refractory ascites or refractory encephalopathy. Our methodology relied on the EDC database using CLD‐related ICD codes, which was then joined to admission data prioritised specificity over sensitivity and may have missed presentations such as patients presenting with gastrointestinal bleeding (K92) who were later diagnosed with varices during admission but had no CLD‐related ED code thus potentially underestimating the true burden of CLD in the ED setting.

## 5 Conclusions

EDs across Queensland experienced an increasing number of presentations by patients with CLD that were associated with high disease burden and complications. A substantial proportion presented to non‐metropolitan EDs, underscoring the importance of equitable access to hepatology expertise. The current epidemiology highlights the need for coordinated care models, incorporating risk stratification and care pathways within EDs, to improve outcomes and manage resource demands.

## Author Contributions

**Akmez Latona:** conceptualization, data curation, formal analysis, funding acquisition, investigation, methodology, project administration, resources, validation, visualisation, writing – original draught. **Alan Ho:** data curation, formal analysis, validation, visualisation, software. **Biswadev Mitra**, **Katherine Stuart**, **Patricia Valery:** supervision, writing – review and editing.

## Funding

This research was supported by a grant from the Emergency Medicine Foundation Australasia (EMLE‐233R38‐2022‐LATONA).

## Conflicts of Interest

The authors declare no conflicts of interest.

## Supporting information


**Figure S1:** Poisson regression–modelled annual CLD‐related ED presentations from 2016 to 2023. Increasing trends noted for total CLD (IRR 1.02, *p* < 0.001), cirrhosis (IRR = 1.0155, *p* = 0.001) and non‐cirrhosis cohorts (IRR = 1.07, *p* < 0.001). Error bars represent 95% confidence intervals.

## Data Availability

The de‐identified data analysed in this study are not publicly available due to ethics and data governance restrictions. Access to these data is subject to approval by the relevant data custodians and ethics committees.
