# Supplementary material for: Epidemiology of Patients With Chronic Liver Disease Presenting to Emergency Departments in Australia
Source: Emerg Med Australas. 2026 Jul 16;38(4):e70310. doi: 10.1111/1742-6723.70310 (PMC13375589; doi:10.1111/1742-6723.70310)
Supplement: Supplementary file 1 — Figure S1: Poisson regression–modelled annual CLD‐related ED presentations from 2016 to 2023. Increasing trends noted for total CLD (IRR 1.02, p < 0.001), cirrhosis (IRR = 1.0155, p = 0.001) and non‐cirrhosis cohorts (IRR = 1.07, p < 0.001). Error bars represent 95% confidence intervals. [file EMM-38-0-s001.docx]

## **SUPPLEMENTARY MATERIAL**

Supplementary Figure 1: Poisson regression–modelled annual CLD-related ED presentations from 2016 to 2023. Increasing trends noted for total CLD (IRR 1.02, p<0.001), cirrhosis (IRR=1.0155, p=0.001) and non-cirrhosis cohorts (IRR=1.07, p<0.001). Error bars represent 95% confidence intervals.


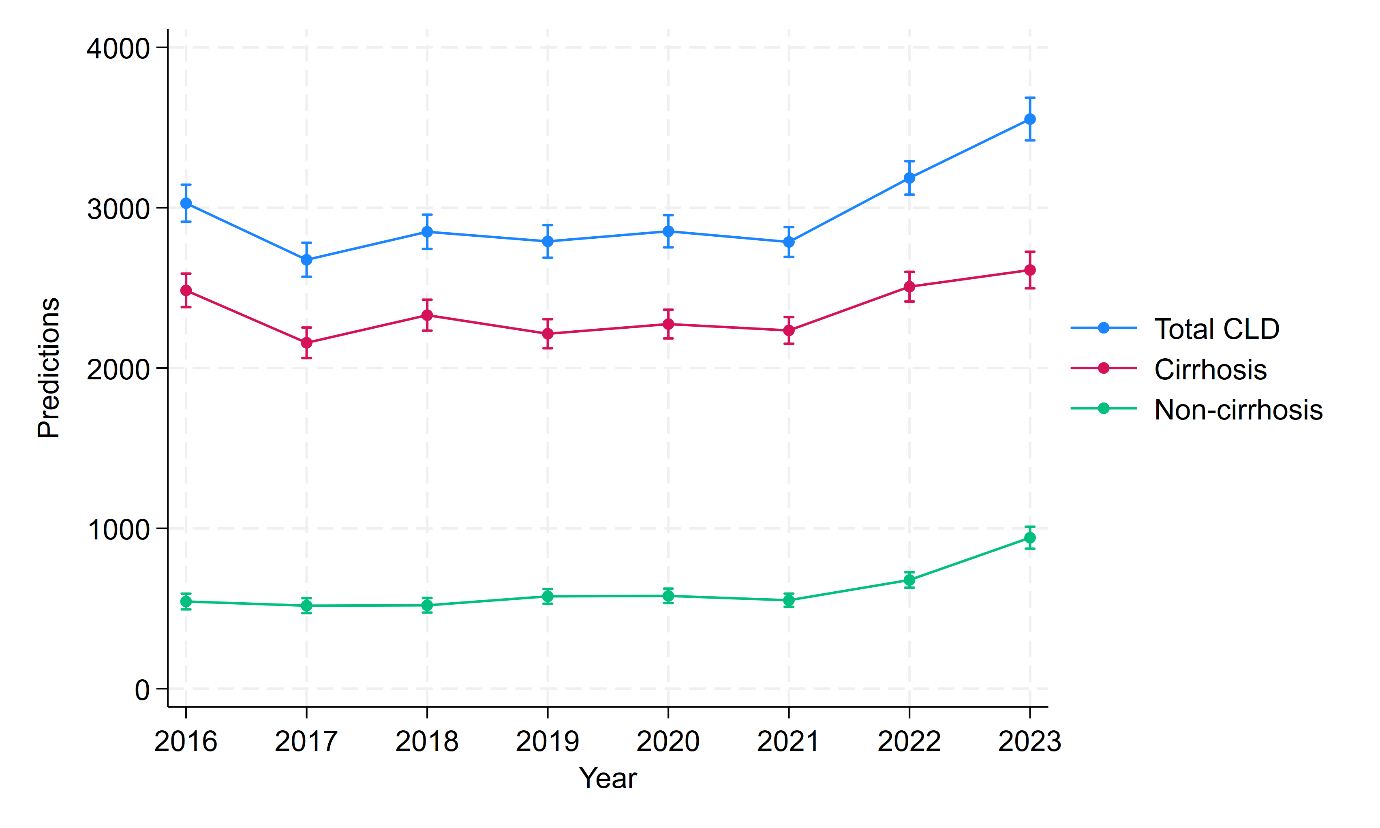


Chronic Liver Disease (CLD) Cohort:

List of International Classification of Diseases, 10th Revision, Australian Modification (ICD-10-AM) codes used to define patients with CLD in the Emergency Data Collection (EDC) and Queensland Health Admitted Patient Data Collection (QHADPC) databases: gastroesophageal varices with/without bleeding (I85.0, I85.9, I86.4, I98.3, I98.2), Liver cell carcinoma (C22), alcohol related liver disease (K70.0, K70.1, K70.2, K70.3, K70.4, K70.9), toxic liver disease with fibrosis and cirrhosis of liver (K71.7), hepatic failure (K72.1, K72.9), chronic hepatitis (K73.0, K73.1, K73.2, K73.8, K73.9), fibrosis and cirrhosis of liver (K74.0, K74.1, K74.3, K74.4, K74.5, K74.6), other inflammatory liver diseases (K75.2, K75.4, K75.8, K75.9), other diseases of liver (K76.6, K76.8, K76.9), chronic liver failure (U843), hepatorenal syndrome (K76.6) and chronic viral hepatitis (B18). In addition, we included ascites (R18) in the EDC if it was accompanied by any of the above ICD codes as “*other diagnosis”* in QHADPC and/or any of cirrhosis related procedure codes (3047603, 3047602, 9033400, 3040600).

Procedures associated with cirrhosis included endoscopic banding of gastric and oesophageal varices (3047603, 33047602), paracentesis (3040600) and transjugular intrahepatic portosystemic shunt (9033400).

Categorisation of CLD cohort:

The CLD cohort was sub-classified into two groups: non-cirrhosis and cirrhosis, with the latter further categorised into acute decompensation and further decompensation.

Amongst the CLD cohort, patients with the following ICD-codes were categorised as cirrhosis: alcoholic fibrosis and sclerosis of liver (K70.2), alcoholic cirrhosis of liver (K70.3), alcoholic hepatic failure (K70.4), chronic hepatic failure (K72.1), fibrosis and cirrhosis of liver (K74.0), primary biliary cirrhosis/cholangitis (K74.3), secondary biliary cirrhosis (K74·4), biliary cirrhosis, unspecified (K74·5), other and unspecified cirrhosis of liver (K74.6), portal hypertension (K76.6), hepatorenal syndrome (K76.7), gastroesophageal varices with/without bleeding (I85.0, I85.9, I86.4, I98.3), hepatocellular carcinoma (C22.0), ascites (R18), peritonitis (K65.0, K65.9), jaundice (R17), encephalopathy (G93.4) and hepatorenal syndrome(K76.7).

Patients with decompensated cirrhosis were identified using the following ICD codes: ascites (R18), variceal bleed (I98.3, I85.0), encephalopathy (G93.4)

Patients with non-variceal gastrointestinal bleeding were identified using K92 in the absence of I98.3 and I85.0

Further decompensation was identified using ICD codes R17 (jaundice), hepatorenal syndrome (K76.7) and peritonitis (K65.0, K65.9)

Portal hypertension was identified using ICD code K76.6, and/or all ICD codes for gastroesophageal varices with and without bleeding (I85.0, I85.9, I86.4, I98.3, I98.2).

CLD was subcategorised according to aetiology with the following ICD codes: viral (B16, B17, B18, B19, Z22.51, Z22.52), MAFLD (K75.8, K76.0), metabolic liver disease ( E83.1, E88.0, E88.8, E88.9), autoimmune liver disease (K74.3, K83.0, K75.4), inflammatory liver disease unspecified (K75.9) and Budd Chiari syndrome (I82.0, K76.5)

Tertiary hospitals included Royal Brisbane & Women's, Princess Alexandra, Sunshine Coast University, Townsville University, Prince Charles, Cairns Base, and Gold Coast University Hospitals.

Metropolitan hospitals included Caboolture, Gold Coast University, Logan, Mater Adult, Princess Alexandra, Queen Elizabeth II, Redcliffe, Redland, Robina, Royal Brisbane & Women's, Sunshine Coast University and Prince Charles Hospitals. Regional hospitals included Bundaberg, Cairns, Gladstone, Gympie, Hervey Bay, Mackay, Maryborough, Nambour, Rockhampton, Toowoomba, Townsville and Ipswich hospitals. The remaining hospitals were categorised as rural/remote.
